# Carbon nanotubes as molecular transporters to study a new mechanism for molecular entry into the cell nucleus using actin polymerization force

**DOI:** 10.1371/journal.pone.0221562

**Published:** 2019-08-22

**Authors:** Shaghayegh Sadr Karimi, Nelly Pante

**Affiliations:** Department of Zoology, University of British Columbia, Vancouver, British Columbia, Canada; University of California Berkeley, UNITED STATES

## Abstract

The transport of macromolecules into the cell nucleus occurs through nuclear pore complexes (NPCs) and is mediated by cellular receptors. Recently, a novel mechanism of nuclear entry, in which actin polymerization provides a propulsive force driving the transport through the NPC, has been proposed. This mechanism is used by the nucleocapsid from baculovirus, one of the largest viruses to replicate in the nucleus of their host cells, which crosses the NPC and enters the nucleus independently of cellular receptors. The baculovirus nucleocapsid contains a protein that hijacks the cellular actin polymerization machinery to assemble actin filaments that propel the nucleocapsid through the host cell cytoplasm. In this study, we functionalized carbon nanotubes by covalently attaching a protein domain responsible for inducing actin polymerization and investigated their nuclear entry. We found that the functionalized carbon nanotubes were able to enter the cell nucleus under permissive conditions for actin polymerization, but not when this process was inhibited. We conclude that the mechanical force generated by actin polymerization can drive cargo entry into the cell nucleus. Our results support a novel force-driven mechanism for molecular entry into the cell nucleus.

## Introduction

Physiological transport of macromolecules from the cytosol into the nucleus occurs through specialized channels called nuclear pore complexes (NPCs) that span the double nuclear membrane [1]. Proteins at the center of the NPC, called nuleoporins or Nups, act as gatekeepers and form a barrier that excludes molecules from the nucleus [2]. However, ions and small molecules with diameters of up to 5 nm [3] can passively diffuse through the NPC. Proteins larger than this diffusion limit and up to 39 nm in diameter [4] may also pass the NPC if they carry specific peptide sequences termed nuclear localization sequences (NLSs) [5]. Cytosolic proteins (termed nuclear import receptors, importins, or karyopherins) bind to NLSs on the protein/cargo and interact with specific Nups to mediate translocation of the cargo through the NPC (reviewed in [6, 7]). The driving force of nuclear import is a gradient of the small GTPase Ran across the nuclear envelope, which switches importins between low- and high-affinity cargo-binding states [8]. However, a newly discovered force-driven mechanism to access the nucleus through NPCs has been recently reported for the pathogen baculovirus [9].

Viruses are opportunistic pathogens that infect their hosts by attacking their cells and hijacking the cellular machinery to replicate and spread infection [10–12]. Many viruses replicate in the nucleus of host cells by using the host cell’s transport machinery (i.e., NPCs, NLSs, importins, GTP, and Ran) to deliver their genomes into the nucleus. However, to avoid interfering with this essential cellular activity, some viruses have evolved divergent strategies to cross the nuclear envelope barrier during infection, which has been only recently discovered (reviewed in [13–15]). One of these new discovered strategies is used by baculoviruses. These DNA viruses have been used extensively as eukaryotic expression vectors for the production of biologically active proteins (reviewed in [16]). However, the detailed molecular mechanism of how baculoviruses deliver their genomes into the cell nucleus has only recently been explored. *Autographa californica* nucleopolyhedrovirus (AcMNPV), the most-studied baculovirus, releases its rod-shaped nucleocapsid (approximately 30×300 nm) into the cytoplasm after endocytosis [17]. With a diameter of about 30 nm, the AcMNPV nucleocapsid is too large to pass through the NPC by passive diffusion. However, high-resolution electron microscopy and electron tomography studies have demonstrated that the AcMNPV nucleocapsid passes lengthwise through the NPC without apparent deformation [18, 19]. This process is independent of importins and the Ran GTPase [9]. Moreover, baculovirus capsid proteins lack NLSs required for normal nuclear import mediated by importins.

Baculoviruses exploit actin-based mobility to transit from the cell periphery towards the nucleus [20]. A nucleocapsid structural protein, VP78/83, hijacks the cellular actin-related protein (Arp) 2/3 complex [21], which is a nucleator of actin polymerization [22]. Cells turn on the actin nucleation activity of the Arp2/3 complex using nucleation-promoting factors, which belong to the Wiskott–Aldrich syndrome protein (WASP) family [23]. All WASP family proteins share a conserved C-terminal VCA (verprolin homology, central, acidic) domain, which is responsible for activating the Arp2/3 complex [24]. Baculovirus VP78/83 is a viral WASP-like protein containing this VCA domain that recruits cellular Arp2/3 to the nucleocapsid, promoting actin polymerization at one of the baculovirus nucleocapsid ends [21, 25]. This creates a “comet tail” that propels the nucleocapsid towards the cell nucleus [20, 26]. It has recently been shown that this actin-based motility drives not only baculovirus migration through the cytoplasm, but also nuclear import of the nucleocapsid [9]. Studies with two different nuclear import assays, one with semi-permeabilized cells and another with isolated nuclei, demonstrated that inhibition of actin polymerization blocked nuclear import of the baculovirus nucleocapsid [9]. Because these assays did not use intact cells, the authors were able to study the role of actin polymerization in nucleocapsid nuclear import, independent of its actin-mediated migration in the cytosol [9]. Based on these studies, the current model for baculovirus nuclear entry is that the nucleocapsid breaches the NPC using the mechanical force of actin polymerization to propel itself through the NPC and enters the cell nucleus without cellular receptors or RanGTP [9].

In this paper we demonstrated the newly discovered force-driven nuclear import mechanism by studying the nuclear uptake of carbon nanotubes (CNTs) conjugated with the VCA domain of WASP to enable CNTs to induce actin polymerization. The results indicate that a cargo with a diameter similar to baculovirus and actin polymerization capability can enter the cell nucleus. Moreover, interfering with the actin polymerization property of the VCA-conjugated CNTs inhibited their nuclear entry. Thus, our results support a novel nuclear transport mechanism, in which actin polymerization is used by cargo as a propulsive force to enter the cell nucleus. This work opens the exciting possibility to develop CNTs as molecular transporter, not only for biological research, but also for gene therapy.

## Results

### Protein conjugation to carbon nanotubes

To develop carbon nanotubes as molecular transporters that mimic the baculovirus nucleocapsid and its actin polymerization-inducing ability, the VCA domain that is common to all WASP family proteins, including baculovirus VP78/83, was conjugated to multi-walled CNTs (MWCNTs) with diameters close to the baculovirus capsid diameter. As single-walled CNTs have diameters up to a few nanometers and the baculovirus capsid has a diameter of 30 nm, MWCNTs were chosen because they can be obtained with diameters in the 1–100 nm range (reviewed in [27]). Protein conjugation onto the surface of MWCNTs was achieved using carbodiimide, according to the protocol described by Jiang et al., 2004 [28]. This protocol takes advantage of the EDC-NHS (EDC: ethyl-dimethylaminopropyl carbodiimide; NHS: N-hydroxysuccinimide) chemistry that is commonly used to crosslink proteins [29] and uses carboxylated-CNTs; thus, the carboxylic groups (–COOH) of CNTs were cross-linked to amino groups in proteins.

**Fig 1:**
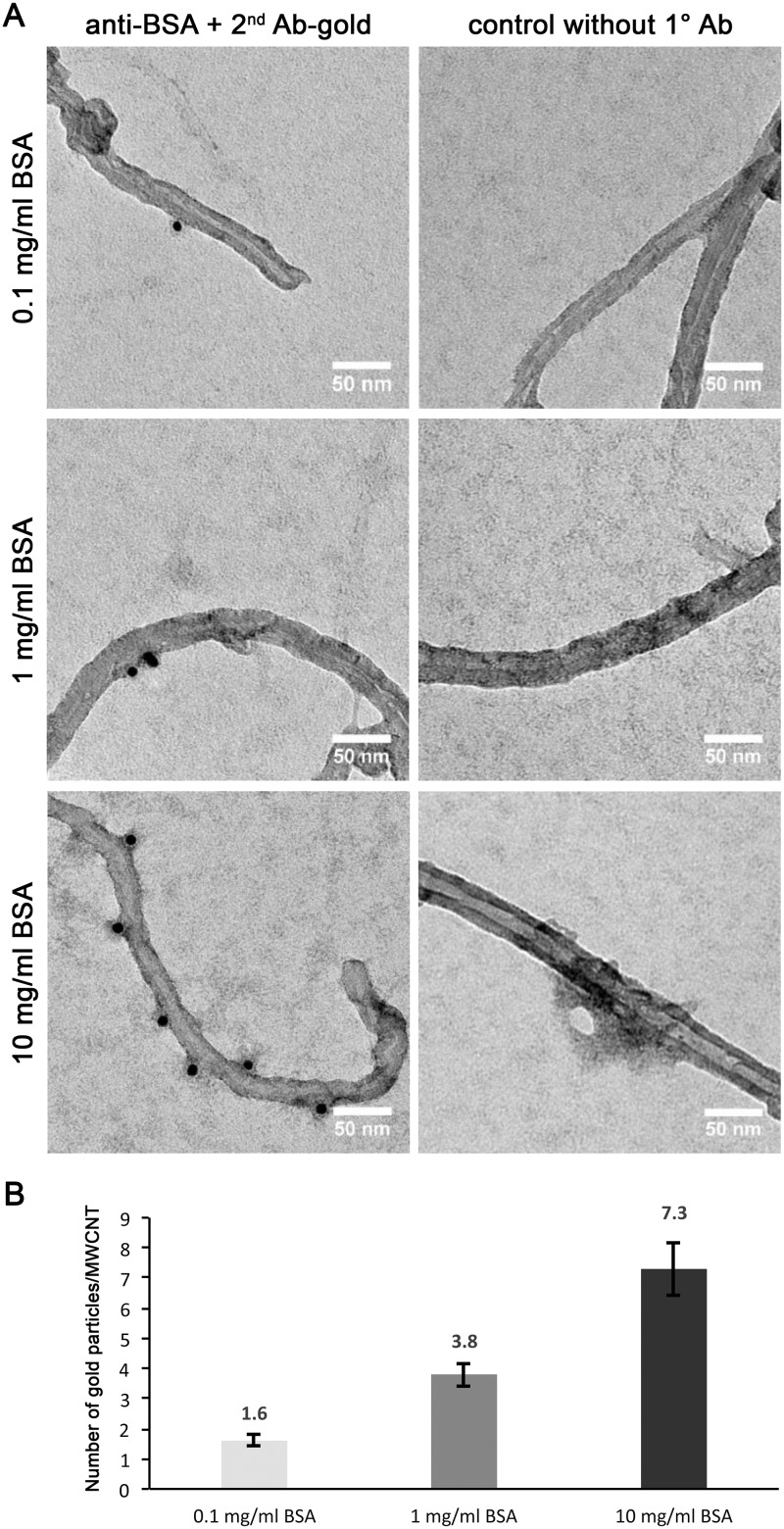
Immuno-gold labeling of MWCNTs conjugated with BSA. (A) Negative staining TEM images of MWCNTs conjugated with BSA at three different protein concentrations and immunolabeled using an anti-BSA antibody and a secondary antibody conjugated to 10 nm gold particles. On the left panels both primary and secondary antibodies were used. The right panels are images of the control experiments in which the immuno-gold staining was performed omitting the primary antibody. (B) Quantification of the number of gold particles on MWCNTs conjugated with BSA. The mean value and standard error of the mean are from 87 MWCNTs for each condition from three independent experiments.

As VP78/83 is located only at one end of the baculovirus capsid, VCA should ideally be attached to only one end of each MWCNT. Unfortunately, we did not have control over the protein conjugation site on MWCNTs, and thus the crosslinking protocol was optimized to yield MWCNT with the least number of proteins attached to it. We first optimized the crosslinking protocol using different concentrations of bovine serum albumin (BSA). BSA attachment to MWCNT was detected by immuno-gold labeling using an anti-BSA antibody, followed by negative staining and transmission electron microscopy (TEM) imaging. As shown in Fig 1, immuno-gold EM confirmed BSA attachment onto the surface of MWCNTs. As expected, the number of gold particles on BSA-MWCNTs was different and increased with protein concentration (Fig 1A, left panels). Moreover, there were no gold particles on MWCNTs for the control experiments in the absence of the primary antibody against BSA (Fig 1A, right panels).

**Fig 2:**
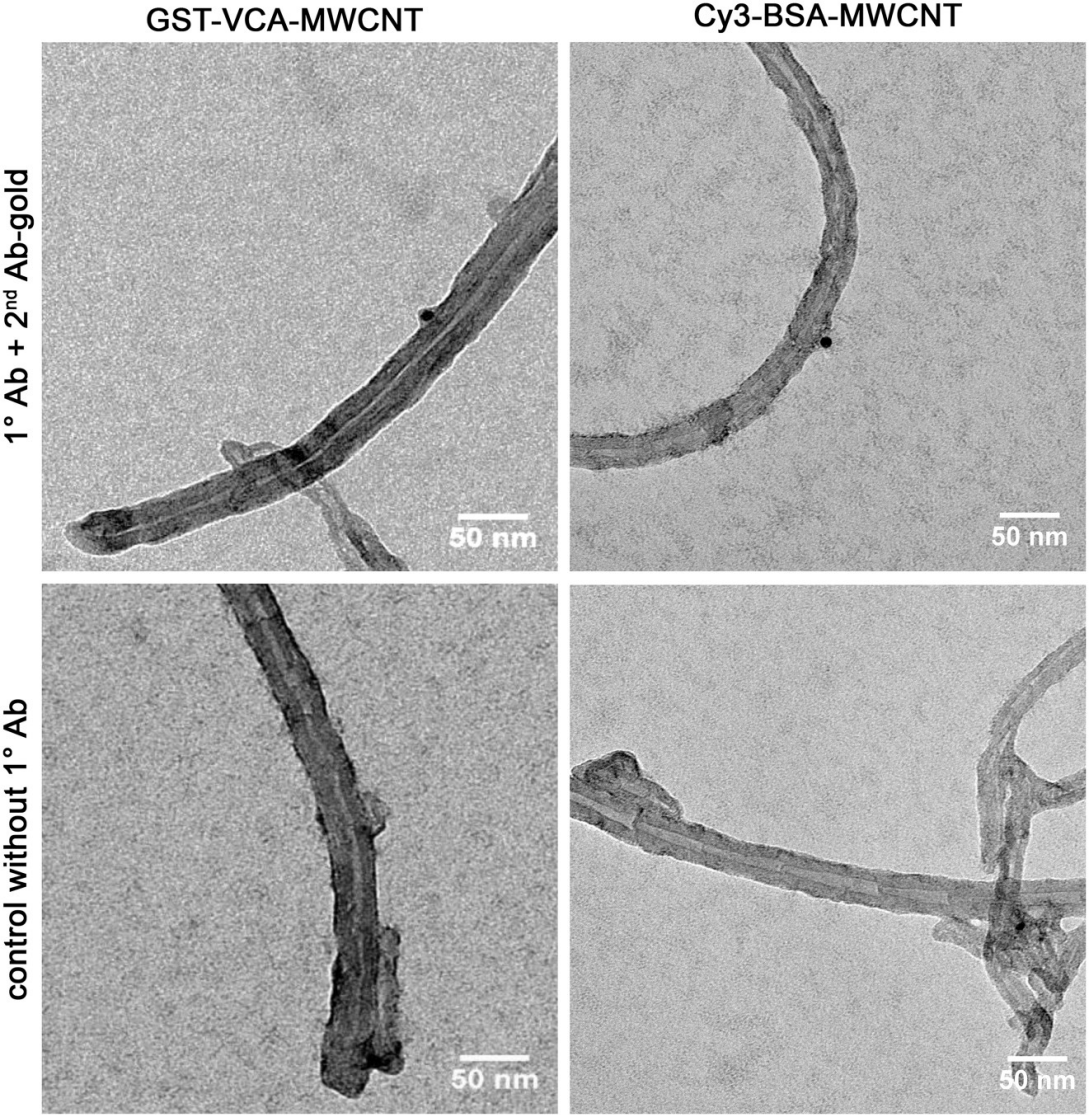
Immuno-gold labeling of MWCNTs conjugated with VCA or with Cy3-BSA. Negative staining TEM images of MWCNTs conjugated with GST-VCA (left) or Cy3-BSA (right) and immunolabeled using primary antibodies against GST or BSA and a secondary antibody conjugated to 10 nm gold particles. The two images at the bottom are from control experiments in which the samples were immuno-gold labeled without the primary antibody.

Quantification of the number of gold particles on MWCNTs showed that when the crosslinking protocol was performed with 0.1 mg/ml of BSA, there were 1.6 ± 0.2 gold particles on each MWCNT (Fig 1B). Thus, 0.1 mg/ml was chosen as the protein concentration to attach VCA to MWCNT. To detect VCA on the MWCNTs, VCA tagged with glutathione S-transferase (GST) was used, which was detected by immuno-gold EM using an anti-GST antibody. Fig 2A is a representative image showing immuno-gold localization of VCA on the surface of an MWCNT. Quantification of the number of gold particles on 100 MWCNTs conjugated with GST-VCA and visualized by TEM after immuno-gold labeling indicated that on average there were 1.4 ± 0.1 gold particles on each MWCNT. Similar to the BSA-MWCNTs, gold particles were located on the VCA-MWCNTs randomly throughout the length of the MWCNT and without any specific pattern.

As a control for the tissue culture cell studies (see below), MWCNTs were conjugated with Cy3-BSA (Fig 2B). Cy3 is a fluorescent dye; thus, MWCNTs conjugated with Cy3-BSA can be directly detected within cells by fluorescence microscopy. We did not conjugate VCA with Cy3 because this interfered with its actin-nucleation activity. Similar to the immuno-gold EM results with BSA-MWCNTs, when using the protein concentration of 0.1 mg/ml, the average numbers of gold particles on Cy3-BSA-MWCNTs were around 1.6 per MWCNT.

### VCA-conjugated MWCNTs can enter tissue culture cells and their nuclei

**Fig 3:**
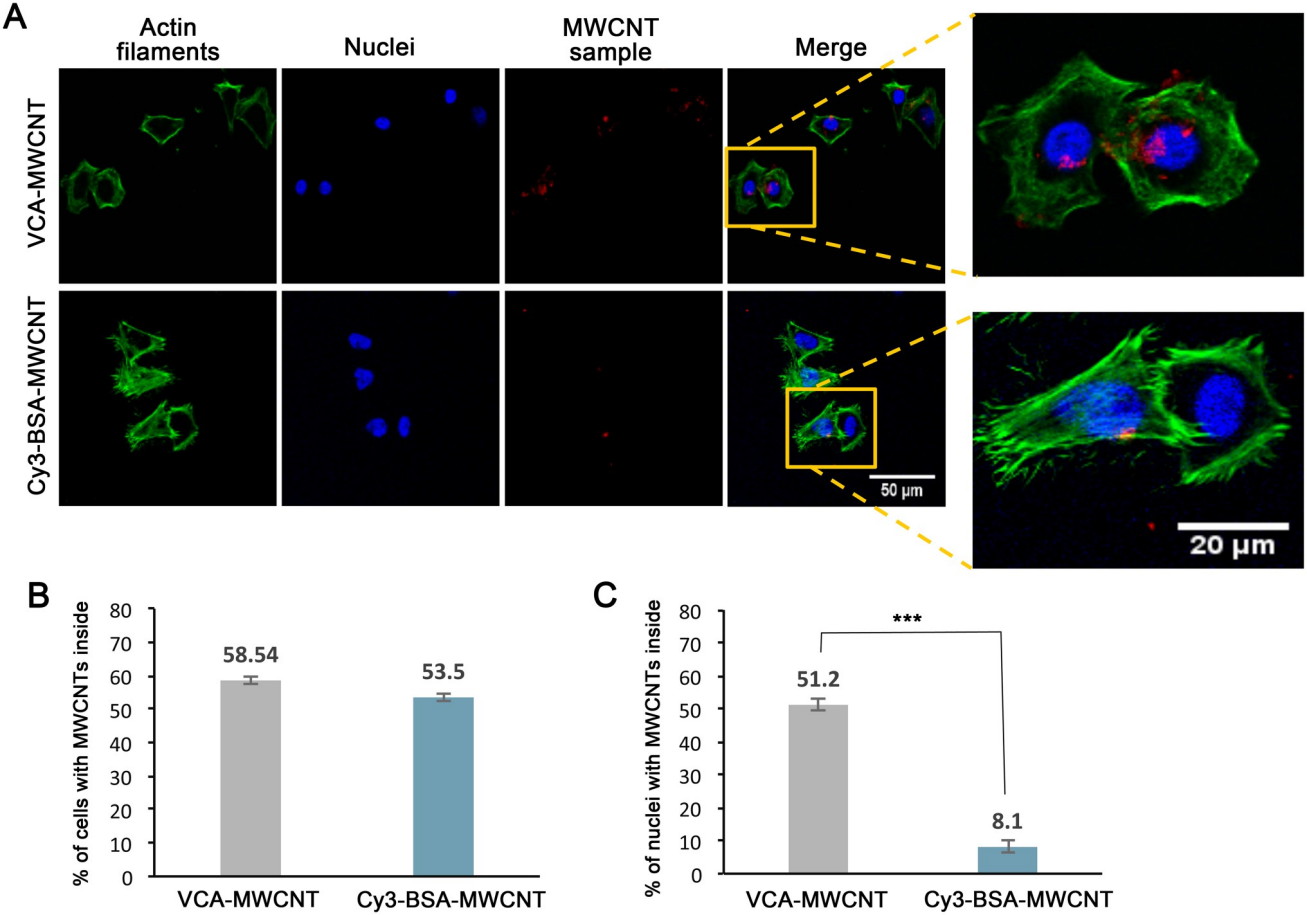
Cellular uptake and nuclear entry of VCA-conjugated MWCNTs in intact HeLa cells. (A) Representative confocal images of HeLa cells incubated with MWCNTs conjugated with GST-VCA or Cy3-BSA (red) as a control. Actin filaments were labeled with Alexa Fluor 488 Phalloidin (green), VCA-MWCNTs were detected by immunofluorescent labeling using an anti-GST antibody (red), and DAPI was used to localize nuclei (blue). Images on the right are enlargements of the associate boxed area. (B) Quantification of cellular uptake of VCA-MWCNTs or Cy3-BSA-MWCNTs in intact HeLa cells for the experiments shown in A. There was no significant difference between the cellular uptakes of the two samples (p = 0.36). (C) Quantification of nuclear uptake of VCA-MWCNTs or Cy3-BSA-MWCNTs in intact HeLa cells for the experiments shown in A. Quantification was performed from images of confocal slices through the middle of the nucleus. Shown in B and C are the mean values ± standard error measured from three independent experiments, n ≥ 90 cells per experiment. Significant differences were determined by chi-square goodness of fit test (*** p<0.001).

Having established a successful protocol for protein conjugation to MWCNTs, we next studied whether MWCNTs conjugated with VCA could enter the nucleus of tissue culture cells. HeLa cells adhered to glass coverslips were incubated with VCA-MWCNTs for 4 hours at 37°C. As a control, cells were incubated with Cy3-BSA-MWCNTs. Cells incubated with Cy3-BSA-MWCNTs were not subjected to immunofluorescence labeling; however, cells incubated with VCA-MWCNTs were immunolabeled using an antibody against the GST tag of VCA. Actin filaments were labeled with Alexa Fluor 488 Phalloidin to locate the cytoskeleton and define the cell periphery. Fig 3A shows confocal images of intact HeLa cells incubated with VCA-MWCNTs or Cy3-BSA-MWCNTs. For both conditions, the red fluorescent signal (representing protein-MWCNTs) was detected inside the cells. Quantifications indicated that cellular entry of MWCNTs conjugated with either Cy3-BSA or VCA was similar (53.5% ± 1.3% and 58.5% ± 1.16% for Cy3-BSA-MWCNT and VCA-MWCNT, respectively; Fig 3B). However, when cells were incubated with VCA-MWCNTs, the number of nuclei with red fluorescent signal was significantly higher than cells incubated with Cy3-BSA-MWCNTs (51.2% ± 1.64% and 8.1% ± 1.7% for VCA-MWCNT and Cy3-BSA-MWCNT, respectively; Fig 3C). These results indicate that MWCNTs conjugated with the VCA domain of WASP are able to enter the cell nucleus.

### Disruption of actin polymerization affects nuclear entry of VCA-MWCNT

**Fig 4:**
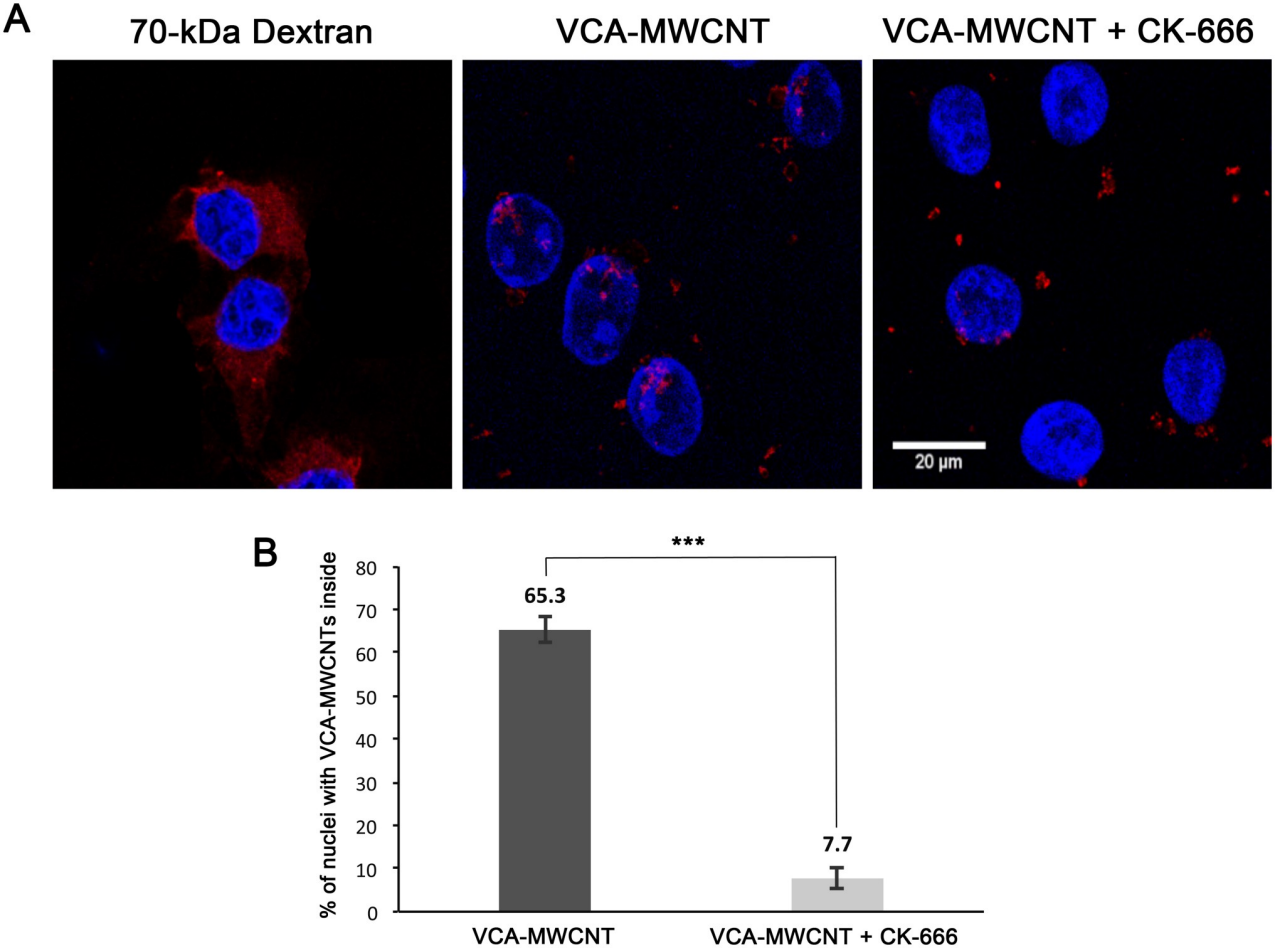
Nuclear import of VCA-MWCNT is impaired by an Arp2/3 inhibitor in semi-permeabilized cells. (A) Confocal images of semi-permeabilized HeLa cells incubated with exogenous cytosol, an energy-regenerating system, and a Texas Red-labeled 70-kDa dextran (left panel) or VCA-MWCNTs in the absence (middle panel) or presence (right panel) of the Arp2/3 inhibitor CK-666. Samples were prepared for immunofluorescent labeling using an antibody against the GST tag of VCA and detected by confocal microscopy. Nuclei were stained with DAPI (blue). The Texas Red-labeled 70 kDa dextran (red) was used to monitor whether the nuclear envelope was disrupted (left image). Confocal z-stacks clearly show the location of the red fluorescence inside the nucleus for cells incubated with VCA-MWCNTs in the absence of CK-666 (S1 Fig), but not in the presence of this drug (S2 Fig). (B) Quantification of the nuclear uptake of VCA-MWCNTs in the absence or presence of CK-666 for the experiments shown in A. Quantification was performed from images of confocal slices that clearly indicated red fluorescence signal inside the nucleus. Shown are the mean values ± standard error measured from three independent experiments, n > 90 nuclei per experiment. Significant differences were determined by chi-square goodness of fit test (***p< 0.001).

The significant difference between the nuclear entry ability of VCA-MWCNT and Cy3-BSA-MWCNT in tissue culture cells (Fig 3C) indicates that, similar to the nuclear import of the baculovirus nucleocapsid [9], the nuclear import of VCA-MWCNT depends on actin polymerization. To test this possibility, the nuclear import of VCA-MWCNT was studied under conditions that disrupted actin polymerization in the presence of an Arp2/3 inhibitor. For these experiments, HeLa cells with digitonin-permeabilized plasma membranes were used. Digitonin is a mild detergent that selectively permeabilizes the plasma membrane without disrupting the nuclear envelope [30]. In this way, the effect of Arp2/3 inhibition on the nuclear import, and not the intracellular trafficking, of VCA-MWCNTs can be observed. Control experiments with a Texas Red-labeled 70-kDa dextran, which is too large to diffuse through the NPC, demonstrated that digitonin did not disrupt the nuclear envelope integrity (Fig 4A).

Once the plasma membrane is permeabilized with digitonin, nuclear transport factors are released from the cell. Thus, to study nuclear import with digitonin-permeabilized cells, exogenous cytosol and an energy-regenerating system must be added to the assay together with a fluorescently labeled cargo [31]. Using this assay, digitonin-permeabilized HeLa cells were incubated with VCA-MWCNTs, exogenous cytosol, and an energy-regeneration system for 30 minutes at 37°C. After incubation, VCA-MWCNTs were detected by immunofluorescence labeling of the GST tag of VCA. As with the purified baculovirus nucleocapsids [9], VCA-MWCNTs entered the nucleus of digitonin-permeabilized cells (Fig 4A). However, when the assay was performed in the presence of CK-666, a drug that inhibits Arp2/3 [32], the number of cells with VCA-MWCNTs in their nuclei was significantly reduced. Quantification showed that 65.3% of semi-permeabilized cells had VCA-MWCNTs inside their nuclei in the absence of CK-666. In CK-666 treated cells, this number decreased to 7.7% (Fig 4B). Thus, similar to nuclear import of the baculovirus nucleocapsid, actin polymerization is involved in the nuclear import of VCA-conjugated MWCNTs.

## Discussion

The baculovirus nucleocapsid uses the host cell’s actin machinery to propel itself into the cell nucleus through the NPC [9]. In this study, we generated a model of baculovirus nuclear import by conjugating the VCA domain of WASP to MWCNTs with diameters similar to the baculovirus nucleocapsid. These VCA-conjugated MWCNTs served as molecular transporters and entered the nucleus of intact tissue culture cells and semi-permeabilized cells. This is in contrast to the corresponding control sample, Cy3-BSA-MWCNT, which did not enter the nucleus of these cells. Thus, attachment of an actin nucleation-promoting factor provides MWCNTs with the potential to enter the cell nucleus.

By inhibiting Arp2/3-mediated actin polymerization in semi-permeabilized cells, we also demonstrated that the nuclear import of VCA-MWCNTs depends on Arp2/3-mediated actin polymerization. The use of semi-permeabilized cells allowed us to separate the two processes that involve actin polymerization, cytoplasmic transport and nuclear import of the VCA-MWCNTs. Results of the experiments with digitonin-permeabilized cells confirm Arp2/3 involvement in the nuclear import of VCA-MWCNTs and further solidify the conclusion that VCA-induced actin polymerization on the MWCNT drives nuclear entry of the VCA-MWCNT.

Our results with VCA-MWCNTs provide evidence for a new nuclear import mechanism by which cargo uses actin-dependent forces to enter the cell nucleus. Although this mechanism could function in special cases, such as nuclear entry of the baculovirus nucleocapsid and nuclear import of artificial cargoes, including VCA-MWCNTs, it has recently been shown that nuclear transport can be regulated by force (reviewed in [33, 34]). For example, nuclear import of the transcription factor YAP/TAZ, a major regulator of cell growth and proliferation, is affected under conditions that influence actin cytoskeleton integrity [35, 36]. Moreover, it has recently been demonstrated that YAP/TAZ nuclear import is increased by direct application of force to the nucleus using an atomic force microscopy cantilever [37]. Remarkably, the nuclear import of YAP/TAP and the nuclear uptake of several other proteins and high molecular weight dextrans (which are normally excluded from the nucleus) were enhanced by force [37]. Thus, the newly discovered, force-induced nuclear import mechanism is a general cellular pathway commonly used under certain physiological conditions. This mechanism could be particularly prominent in migratory cells, which apply cytoskeleton-exerted forces on the nucleus.

Although this and other studies have clearly identified actin-mediated forces as important players in nuclear import, the force-induced rearrangement that must occur in the central channel of the NPC to allow cargo transport remains to be determined. Nevertheless, a recent electron microscopy analysis found that the size of the NPC central channel increased under conditions in which force was applied to the nucleus [37]. Further experiments will be crucial in unraveling the exact mechanisms by which a force applied directly at the NPC (similar to the baculovirus nucleocapsid) or on the nuclear membrane opens the central transport barrier of the NPC. Results of future experiments will potentially explain the nature of the Nup barrier at the center of the NPC and examine whether it resembles a diffuse polymer brush, a dense hydrogel, or a combination of these two as it has been proposed (reviewed in [38]).

In the past decade CNTs have been considered as promising drug delivery vehicles due to their unique properties such as lightness, strength, large surface area, and enhanced permeability (reviewed in [39]). However, one of the major limitations of non-viral transfection agents is the inability to transport the recombinant nucleic acids into the nucleus of the target cell [40]. Thus, if CNTs were equipped or coupled with an effective nuclear transport strategy, they would be ideal for gene transfer. Our results indicated that VCA-conjugated CNTs efficiently enter the cell nucleus. Thus, these VCA-CNTs could be developed as a biomimetic gene delivery vehicle that uses actin-based propulsion for nuclear import. Therefore, our work opens the exciting possibility to develop CNTs as gene delivery vehicles that use force-drive mechanism for nuclear import.

In summary, our CNT results indicate that a cargo can enter the nucleus using the mechanical force generated by actin polymerization. Thus, in addition to the two established nuclear transport mechanisms (i.e., passive diffusion of small molecules and active receptor-mediated translocation of large molecules), a third force-mediated mechanism exists to allow entry into the cell nucleus. Understanding this force-dependent nuclear import mechanism will potentially increase understanding of how the NPC functions.

## Material and methods

### Cells and chemicals

HeLa cells were cultured at 37°C and 5% CO_2_ in Dulbecco’s modified Eagle medium (DMEM, Sigma Aldrich) supplemented with 5% fetal bovine serum (FBS, Sigma Aldrich), 1% penicillin-streptomycin (Cellgro), 2 mM L-glutamine (Thermo Fisher Scientific), and 1 mM sodium pyruvate (Thermo Fisher Scientific).

Bovine serum albumin (BSA), 2-(N-morpholino) ethane sulfonic acid (MES) buffer, normal goat serum (NGS), and digitonin were purchased from Sigma-Aldrich Corporation. N-hydroxysuccinimide (NHS), 1-ethyl-3-(3-dimethylaminopropyl) carbodiimide (EDC), and all other chemicals were purchased from Thermo Fisher Scientific.

### BSA-Cy3

BSA was tagged with the Cy3 bis-reactive fluorescence dye (Cy3; GE Healthcare) according to the manufacturer’s instructions. Briefly, BSA at a concentration of 0.4 mg/ml and Cy3 were mixed in the presence of 0.1 M sodium bicarbonate (pH 9.3) for 45 minutes at room temperature. Then, to remove the excess of Cy3, the mixture was washed four times with phosphate-buffered saline (PBS) in a 30-kDa cut off centrifugal filter unit (Millipore Ltd., catalog number: UFC803024).

### Carbon nanotubes

Purified carboxylated multiwalled carbon nanotubes (MWCNT-COOH) were purchased from Nanostructured & Amorphous Materials, Inc., (Stock No: 1257YJF). This product was over 95% pure and had 1.17–1.29 weight percent content of carboxyl group. According to the manufacturer, these MWCNT-COOH were 0.5–2 μm long with 20–30 nm outside diameter and 5–10 nm inner diameter. Our analysis of these MWCNTs by transmission electron microscopy (TEM) after negative staining using our protocol described below, showed that the average diameter of these MWCNT was 23.49 ± 0.95 nm (n = 40), which is in the range reported by the manufacturer.

### Protein conjugation to MWCNTs

The protocol described by Jiang et al. (2004) was adapted to covalently bind proteins to carboxylated-MWCNTs [28]. This protocol had two main steps: in the first the carboxylic acid groups on MWCNTs were converted to active ester groups via diimide-activation using the carbodiimide EDC in the presence of NHS; this step yields estered-MWCNTs. In the second step, the active esters on MWCNTs were reacted with amine groups on proteins.

For the first step, 2 mg of MWCNT-COOH was suspended in 5 ml deionized water by sonication for 15 minutes and then 1 ml MES buffer (500 mM, pH 6.1) and 2.3 ml of NHS aqueous solution (430 mM) were added to the MWCNT solution and mixed. Next, 1.2 ml of a fresh made 50 mM EDC aqueous solution was added and the mixture was stirred for 30 minutes at 400 rpm on a platform shaker. The suspension was then washed five times with MES buffer (50 mM, pH 6.1); each time the mixture was centrifuged at 3500 rpm for 5 minutes to remove excess EDC, NHS, and by-products. Finally, the pellet containing estered-MWCNTs was resuspended in 10 ml of 50 mM MES buffer.

For the second step, 100 μl of estered-MWCNT was placed in a 0.5 ml Eppendorf tube and the tube was sonicated in an ice-water bath for 30 minutes. Then, 10 μl of protein at different concentrations (see below) was added and the solution was stirred on a platform shaker at 400 rpm for 1 hour. To separate protein-conjugated MWCNTs from unbounded protein, the solution was washed three times with 50 mM MES buffer; the mixture was centrifuged at 14,000 rpm for 10 minutes each time. After the last centrifugation, the protein-MWCNT pellet was resuspended in a 50 mM Tris buffer (pH 8) and incubated for 10 minutes while shaking on a platform shaker at 400 rpm to deactivate all active ester groups on the MWCNTs. Next, protein-MWCNTs were washed 3 times with ice-cold PBS by centrifuging the mixture at 14,000 rpm for 10 minutes each. Protein-MWCNTs were then resuspended in PBS and used for experiments immediately or kept at 4°C to be used the following day.

Three proteins were covalently bound to estered-MWCNT: BSA, Cy3-BSA (prepared as described above), and the VCA domain of human WASP tagged with glutathione S-transferase (GST) on its N-terminus (GST-VCA; Cytoskeleton Inc., catalog number: VCG03-A). The protein concentrations used for conjugation to MWCNT were: 10 mg/ml, 1 mg/ml, and 0.1 mg/ml for BSA and 0.1 mg/ml for Cy3-BSA and GST-VCA. The estered-MWCNTs conjugated with these three proteins are hereafter abbreviated as BSA-MWCNT, Cy3-BSA-MWCNT, and VCA-MWCNT.

### Immuno-gold labeling, negative staining, and transmission electron microscopy

To confirm the successful conjugation of proteins to MWCNTs, proteins were labeled with antibodies against the protein and secondary antibodies conjugated to 10 nm gold particles. First, a 10-μl drop of protein-MWCNT was placed on a copper EM grid that was freshly coated with 2% parlodion and carbon. All the subsequent steps were performed while the grids were placed in a wet chamber. After 15 minutes, each grid was incubated facing down on a 15-μl drop of a blocking solution (50 mM MES buffer containing 1% BSA, when blocking MWCNT conjugated with the GST-VCA, or 0.5% NGS, when blocking MWCNT conjugated with BSA or BSA-Cy3) placed on a clean parafilm surface, two times for 5 minutes each. After blocking, grids were washed 4 times on 50-μl drops of 50 mM MES buffer (pH 6.1) for 1 minute in total; each time the grid was placed on top of a drop and gently swiped on that drop. Grid washings in all other steps were performed in this way. After washing, the grids were incubated with 15 μl drops of a primary antibody diluted in a washing buffer (blocking solution diluted 10 times) for 1 hour. Next, grids were washed 3 times with 50-μl drops of the washing buffer for 5 minuets each, followed by incubation with a 15-μl drop of a secondary antibody conjugated with 10 nm gold particles, diluted in the washing buffer, for 1 hour. After 3 washings with the washing buffer and 3 washings with PBS for 5 minutes each, samples were fixed with 1% glutaraldehyde (Sigma Aldrich) in PBS for 5 minutes. Then, the negative staining was performed by adding 10 μl of 1% uranyl acetate (Ted Pella Inc.) to the grid. After one minute, the uranyl acetate was blotted off using a piece of filter paper and the grid was allowed to air dry.

To detect GST-VCA on the MWCNTs, a primary mouse monoclonal antibody against GST (GenScript, catalog number: A00865, dilution 1:500) and a secondary goat anti-mouse antibody conjugated with 10 nm gold particles (Ted Pella Inc., catalog number: 15751, dilution 7:500) were used. To detect BSA, a primary rabbit polyclonal anti-BSA antibody (Life Technologies, catalog number: A-11133, dilution 1:500) and a secondary goat anti-rabbit antibody conjugated with 10 nm gold particles (Ted Pella Inc., catalog number: 15726, dilution 7:500) were used. For all experiments, control samples were prepared in the absence of primary antibodies.

Samples were visualized using a FEI Tecnai G2 Spirit TEM operated at an accelerated voltage of 120 kV. Micrographs were digitally recorded using an Eagle 4k CCD camera (FEI).

### Incubation of tissue culture cells with protein conjugated MWCNTs

HeLa cells seeded on 35 mm glass coverslips at about 75% confluence were incubated with VCA-MWCNTs or Cy3-BSA-MWCNTs for 4 hours at 37°C and 5% CO_2_. Next, cells were washed 3 times with PBS. Cells incubated with MWCNT conjugated with GST-VCA were prepared for indirect immunofluorescence microscopy to detect GST with an antibody as indicated below. Cells incubated with Cy3-BSA-MWCNT were kept on PBS. At the end, all coverslips were mounted on glass slides at the same time as indicated below.

### Nuclear import assay with semi-permeabilized HeLa cells

HeLa cells seeded on glass coverslips at about 75% confluence were rinsed with ice-cold import buffer (IB: 20 mM HEPES, pH 7.4, 110 mM potassium acetate, 1 mM EGTA, 5 mM sodium acetate, 2 mM magnesium acetate, 2 mM dithiothreitol) and then permeabilized for 3 minutes with 20 μg/ml ice-cold digitonin in IB. After 3 washings with ice-cold IB, each for 10 minutes, coverslips were incubated with IB containing VCA-MWCNTs or Cy3-BSA-MWCNTs in the presence of an energy-regeneration system (0.4 mM ATP, 0.45 mM GTP, 4.5 mM phosphocreatine and 18 U/mL phosphocreatine kinase; all chemicals from Sigma Aldrich), 20% rabbit reticulocyte lysate (Promega) and Halt protease inhibitor cocktail (Thermo Fisher Scientific, catalog number: 78430, dilution 1:100) for 45 minutes at 37 °C. As a control for the intactness of the nuclear envelope, semi-permeabilized cells were incubated with IB containing a 70-kDa dextran coupled to the fluorescent dye Texas Red (Life Technologies), which is too large to diffuse through the NPC.

Next, cells were washed with IB three times, each for 10 minutes. Cells incubated with fluorescent dextran or Cy3-BSA-MWCNTs were kept in PBS, and cells incubated with VCA-MWCNTs were prepared for immunofluorescence microscopy as indicated below. At the end, all coverslips were mounted on glass slides at the same time as indicated in below.

To study the effects of inhibiting actin polymerization mediated by the Arp2/3 complex on the nuclear import of VCA-MWCNTs, cells were incubated with 1 mM CK-666, a cell-permeable Arp2/3 inhibitor (Millipore Ltd.), for 1 hour at room temperature prior to incubation with samples. CK-666 was also added to the import mixture at a concentration of 1 mM.

### Indirect immunofluorescence labeling

Glass coverslips with intact HeLa cells or digitonin-permeabilized cells incubated with VCA-MWCNT and assayed as indicated above were fixed with 3% paraformaldehyde (Polysciences Inc.) in PBS for 10 minutes. Samples were gently washed with PBS followed by 5 minutes of permeabilization with 0.2% Triton X-100 (Sigma Aldrich). The coverslips were then incubated with the blocking buffer containing 10% NGS in PBST (PBS and 0.1% Tween 20), at room temperature for 20 minutes. After blocking, samples were incubated with primary antibodies diluted in PBS containing 1% BSA for 1 hour at room temperature. Excess amount of primary antibody was removed by washing three times with PBS. Next, the samples were incubated with an appropriate fluorophore-conjugated secondary antibody, diluted in PBS containing 1% BSA, for 40 minutes at room temperature. Coverslips were then washed three times at 5 minutes intervals with PBS. At last, coverslips were mounted with ProLong Diamond antifade mountant with DAPI (Invitrogen).

To detect GST-VCA, a mouse monoclonal antibody against GST (GenScript, catalog number: A00865, dilution 1:500), and a goat anti-mouse Alexa Fluor 568 (Invitrogen, catalog number: 1793903, dilution 1:600) were used as primary and secondary antibodies, respectively. For the experiments with intact cells, Alexa Fluor 488 Phalloidin (Thermo Fisher Scientific, catalog number: A12379) was added to the mix of secondary antibodies in a dilution of 1:200 to label filamentous actin.

### Confocal microscopy, image analysis, and quantifications

Fluorescence microscopy images of HeLa cells or digitonin-permeabilized cells assayed as indicated above were acquired using a confocal laser-scanning microscope (Olympus Fluoview FV1000 and Leica SP5). Confocal images shown in this paper are representatives of at least three independently repeated experiments.

Statistical data analysis was performed using RStudio software. To compare the significance of differences between experimental results and hypothesized values, two tailed Chi-square Goodness-of-Fit test was used. A significant level of 0.05 was established as a maximum level to consider a difference statistically significant. All the presented fluorescence images are representative of at least 3 independent experiments. Quantification reported corresponds to the mean ± standard error from three independent experiments.

## Supporting information

S1 Fig Confocal z-stack of digitonin permeabilized HeLa cells incubated with exogenous cytosol, an energy-regenerating system, and VCA-MWCNTs. (TIF)

S2 Fig Confocal z-stack of digitonin permeabilized HeLa cells incubated with exogenous cytosol, an energy-regenerating system, and VCA-MWCNTs in the presence of the Arp2/3 inhibitor CK-666. (TIF)
