# Supplementary figures and images for: Carbon nanotubes as molecular transporters to study a new mechanism for molecular entry into the cell nucleus using actin polymerization force
Source: PLoS One. 2019 Aug 22;14(8):e0221562. doi: 10.1371/journal.pone.0221562 (PMC6705785; doi:10.1371/journal.pone.0221562)

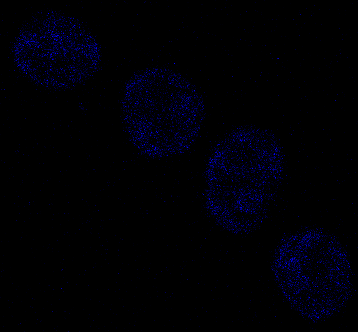

Supplement: S1 Fig — (TIF) [file pone.0221562.s001.tif]

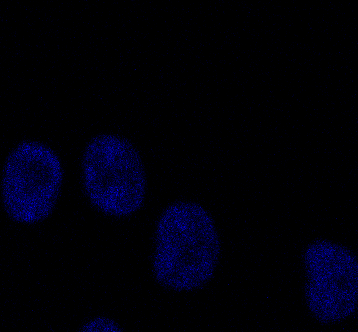

Supplement: S2 Fig — (TIF) [file pone.0221562.s002.tif]
